# Food Insecurity among Low-Income Food Handlers: A Nationwide Study in Brazilian Community Restaurants

**DOI:** 10.3390/ijerph18031160

**Published:** 2021-01-28

**Authors:** Ingrid C. Fideles, Rita de Cassia Coelho de Almeida Akutsu, Rosemary da Rocha Fonseca Barroso, Jamacy Costa-Souza, Renata Puppin Zandonadi, António Raposo, Raquel Braz Assunção Botelho

**Affiliations:** 1 Department of Food Science, School of Nutrition, Federal University of Bahia, Salvador 40110-150, Brazil; ingridfideles@gmail.com (I.C.F.); rosemary.fonseca2017@gmail.com (R.d.R.F.B.); jamacy@ufba.br (J.C.-S.); 2 Department of Nutrition, Faculty of Health Sciences, University of Brasilia, Brasilia 70910-900, Brazil; renatapz@yahoo.com.br (R.P.Z.); raquelbabotelho@gmail.com (R.B.A.B.); 3 CBIOS (Research Center for Biosciences and Health Technologies), Universidade Lusófona de Humanidades e Tecnologias, Campo Grande 376, 1749-024 Lisboa, Portugal

**Keywords:** Brazil, community restaurants, food handlers, food insecurity, low-income

## Abstract

This study aims to evaluate food insecurity (FI) among Brazilian Community restaurant food handlers and its associated factors. This cross-sectional study was performed with a representative sample of 471 food handlers working in community restaurants (CR) from all Brazilian regions. Participants are mostly female (62.2%), ≤40 years old (67.7%), with a partner (52.0%), and with up to eight years of education (54.1%). Predictors of participants’ socioeconomic status and CR geographic location are associated with the household food insecurity categories (*p* < 0.05). The predictors of socioeconomic conditions are associated with mild and moderate/severe FI category. Workers with less education are twice as likely to belong to the category with the highest FI severity. Lower per capita household income increased the chances of belonging to the mild insecurity category by 86%. It more than doubled the chance to be in the category of moderate/severe insecurity. Predictors of health status, lifestyle, and work are not associated with any multinomial outcome categories. However, working in the South, Southeast, or Midwest regions of Brazilian decreased the chances of belonging to one of the FI categories, with significance only for the mild category. Variables that show an association for this population are per capita household income for the different levels of FI and the CR region for mild FI. A high prevalence of FI in this population points to the need for more studies with low-income workers to prevent FI and its health consequences.

## 1. Introduction

Food insecurity is a global public health problem, affecting more than 2 billion people worldwide [1]. Food security is a basic need covering access to safe, sufficient, and proper nutritional food [2,3]. Among adults, food insecurity is related to the high prevalence of chronic health problems that compromise this population’s quality of life and longevity. It is strongly linked to economic vulnerability [1,4]. In Brazil, the estimation is that more than 14 million households have some food insecurity level [5]; additionally, people eat more outside their homes because they have less time to prepare meals. In Brazil, to the low-income population, eating out tends to represent an intake of cheaper and fast snacks, usually representing inadequate nutrient intake that influences their health outcomes increasing the risk of chronic diseases and nutrient deficits [2,6,7,8].

In Brazil, people with no or limited access to adequate, safe, and nutritional food are characterized in food insecurity situations (FIS) [5]. FIS can generate fear of the inability to obtain food (influencing quantity or quality of food choice) or generate hunger in the most severe cases due to the scarcity of food at home [5]. The FIS determinants can present social, economic, and political nature, affecting populations’ health. The living and working conditions of individuals and population groups are related to their health situation [9]. Among the factors associated with FIS, mainly in the moderate and severe categories of FI, income, and weight excess have been highlighted [10]. Women in developed countries have higher chances of being overweight and obese when in FIS [11].

Based on this information, the Brazilian Government developed the community restaurant (CR) program as a strategy to face the FIS. CRs were created to offer safe, cheap, and healthy meals to the low-income population [12,13]. The CR Program is one of the programs integrated into the “Fome Zero” network of actions, a social inclusion policy established in 2003 [13]. From the beginning, the Government planned to increase the number of CR distributed throughout the Brazilian territory located in regions with more significant numbers of low-income people, reaching 135 CRs in 2020 [13,14]. The production and distribution of CR involve professionals with different education and income levels [15], highlighting the food handlers that directly produce meals [13]. Among food handlers, it is common to have weight excess [15,16,17,18], low education levels, and low-income [15,19], making them more susceptible to FIS [10] even though they work in places that produce a great amount of food. Godoy et al. [20] studied food insecurity among CR customers. Despite a high percentage of FI (40.6% for males and 43.8% for females), there were no significant associations between FI and Body Mass Index or body fat percentage. There were significant associations between FI and household income and educational levels. There was also an association with Brazilian’s regions among females, FI being worse in the North and Northeast regions.

Few studies evaluated FIS among workers in Brazil [21,22,23] and in the world [24,25,26,27]. However, only three of these studies were performed with professionals working in food service. A study showed a high prevalence of FIS perception among these professionals in Canada compared to other professionals [24]. The two studies conducted in Brazil only evaluated food handlers in a specific region without a nationwide Brazilian representative sample of food handlers [21,22]. Studies with the population of food handlers demonstrate their susceptibility to food insecurity [21,22], to excess weight [22,28], to management in foodservice with a greater focus on the costs involved with the production of meals than with the health of workers [29]. Even though the studies found in Brazil were both developed in the format of case studies [30,31,32], geographically delimited to institutional spaces, such as universities, Brazilian states or municipalities [21,22,23,28,31,32,33], food insecurity is multifactorial and, in CR, we hypothesize that socioeconomic and demographic factors of food handlers are associated with food insecurity. Since the program tends to increase the number of CR in Brazilian territory, more CRs open over the country, possibly resulting in more opportunities to decrease FI among customers and more work opportunities for food handlers. However, these food handlers need to be closely investigated because of their well-being, quality of life, and food security to guarantee motivation to work and meals safely produced. Therefore, the objective was to evaluate nationwide the food insecurity among Brazilian CR food handlers and its associated factors. As it has an exploratory character, the study sought to answer the following question: What are the factors associated with food insecurity among food handlers in Brazilian CR? We expect to provide data allowing to promote interventions to reduce food insecurity among this vulnerable group that works inside foodservices but does not overcome FI inside their families.

## 2. Materials and Methods

### 2.1. Design, Settings, and Participants

This cross-sectional study was performed in Brazilian CR (focused on the low-income Brazilian population offering meals from Monday to Friday). It was conducted according to the Declaration of Helsinki guidelines and approved by the University of Brasília Research Ethics Committee (#037210). Written informed consent was obtained from all participants.

The basis of the sample calculation was the official list of all existing CR throughout the Brazilian territory at the moment of data collection [13]. With CR nationwide selection, food handlers in different Brazilian regions and similar work conditions were evaluated. Researchers were allowed to enter all of the CR with nationwide permission. The restaurant inclusion criteria were to be part of the Brazilian CR program, sign the Institutional Acknowledgement Agreement by the CR responsible, and offer daily more than 500 meals. With more than 500 meals, CR allowed researchers to evaluate many workers, helping to achieve a representative sample. Restaurants that provide less than 500 daily meals are considered small and present fewer food handlers than medium or large restaurants that offer more than 500 daily meals [34]. There were 65 existing CR in Brazil, and all of them met the inclusion criteria.

From the 65 CR, a sampling plan was calculated considering a level of significance (α) of 5% [35] using the “survey select” of the SAS 9.1.3 program (SAS Institute Inc., Cary, NC, USA). Therefore, a minimum of 31 CR existing in each of the five Brazilian regions (North, Northeast, Midwest, South, and Southeast) was randomly selected to be part of the study. The final sample of 36 restaurants was used, respecting the stratification criteria by the Brazilian region. The distribution of randomly drawn CRs was proportional to the number of CR in each region. The researchers visited 36 CR and included them in the sample (4 in North, 10 in Northeast, 1 in Midwest, 15 in Southeast, and 6 in South). All of the working food handlers in the 36 drawn CR were invited to participate in this study (*n* = 1062). Data collection was carried out over four consecutive days to cover the various work schedules to guarantee access to the largest possible number of handlers in each CR. Therefore, from the total of 1062 handlers working on the selected CRs, 970 met the inclusion criteria (e.g., not being pregnant, and workers on vacation or not working due to medical issues). From them, 471 (48.6%) agreed to participate and completed the study.

Some handlers refused to participate in the research because they did not want to stop working or worried about employability. Even though researchers explained that participation would not influence their work, some decided not to participate in the study. A total of 383 individuals was necessary to be a representative sample stratified in the five Brazilian regions according to the number of food handlers in each region. Participants were not compensated for the participation. They were just informed about the importance of the study for their category. The study used a 95% confidence interval and an error of 4%, respecting the minimum handlers’ sample per Brazilian region [35]. Therefore, it was necessary to achieve a minimum of 11% of the handlers from the North, 28% from the Northeast, 3% from the Midwest, 41% from the Southeast, and 17% from the South.

### 2.2. Data Collection

Trained researchers performed data collection using standardized instruments to identify socio-demographic characteristics [36] and the Brazilian Food Insecurity Scale (EBIA/BFIS) [37]. The socio-demographic variables were gender, age, educational level, per capita household income, marital status, smoking status, participation in a governmental program, and how many years or months the individual has worked in the CR. The presence of diagnosed non-communicable diseases (NCDs) (by a physician) was self-reported. The information was recorded in a specific form showing the presence or absence of one or more than one of the following NCDs: Systemic arterial hypertension (SAH), type 2 diabetes mellitus (DM), and others (cancer, dyslipidemia, cardiovascular diseases, respiratory diseases, depression). The self-reported NCD data was used because population studies widely use this method for its convenience and economy [36,38].

The individuals who agreed to participate signed the consent form after receiving information about the research. Before participants’ lunch in a reserved room, weight and height were measured with a Plenna^®^ (São Paulo, Brazil) weighing scale (150 kg) and a stadiometer (220 cm). Participants had to take off their shoes and coats. After that, the body mass index (BMI) was calculated [39]. The anthropometric status based on body mass index classification [39] was dichotomized on weight excess (0. No; 1. Yes). The cut-off point used to indicate excess weight was a BMI value ≥ 25 kg/m^2^, which covers both the category of overweight and obesity.

### 2.3. Dependent Variable

The study’s outcome, household food security situation (HFSS), was obtained through EBIA/BFIS adapted and validated for the Brazilian population [40,41]. The EBIA/BFIS seeks to assess the perception and experience of household residents’ hunger in the three months before the instrument application. The positive answers to the 14 questions of the instrument categorize the level of food security/insecurity (considering the age of the residents) in food security (0 points); mild food insecurity (1–5 points in the presence of residents <18 years old, or 1–3 points in the absence of residents <18 years old); moderate food insecurity (6–9 points in the presence of residents <18 years old, or 4–5 in the absence of residents <18 years old) and, severe food insecurity (10–14 points in the presence of residents <18 years old, or 6–8 points in the absence of residents <18 years). Based on the methodology adopted by Panigassi et al. [42], for this study, the HFSS categories were grouped into food security, mild food insecurity, and moderate/severe food insecurity.

### 2.4. Statistical Analysis

The data were analyzed with the STATA 15.0^®^ (StataCorp LP, College Station, TX, USA), using frequencies to describe the categorical variables and Pearson’s chi-square test (χ^2^) to identify associations (*p*-value < 0.05).

The outcome with three categories of HFSS, multinomial (polytomous) logistic regression models were used. They are applicable for outcomes with three or more levels [43]. For this study, the category “food security” was chosen as a reference. In this type of analysis, the logistic model will have two logit functions: the ratio between Y = 1 and Y = 0 and the ratio between Y = 2 and Y = 0, with Y = 0 as the referent. The numbers 0, 1, and 2 represent the food security status classification for statistical analysis—(0) Food Security—the household has regular and permanent access to food in sufficient quantity and quality without compromising access to other needs; (1) Mild food insecurity—at this level there is uncertainty regarding access to food in the future, with a change in the quality of food, but without compromising the amount of food; (2) includes the levels of moderate food insecurity and severe, in which there is already a quantitative reduction in access to food, causing changes in the dietary pattern of residents at home. These categories were grouped to enable comparison with population data given that national food insecurity assessment studies [44] and international [45] present their analyses considering the grouped on these two levels.

Bivariate multinomial regression models, with HFSS as the dependent variable, were applied to all predictor variables. This stage helped to understand the initial associations of the determinant factors for the HFSS of this population and the magnitude of each predictor’s effect by calculating the gross Odds Ratio (OR) and their respective 95% Confidence Intervals (CI). The predictor variables were included in the multivariate model based on two assumptions. The first assumption was the theoretical basis underlying the HFSS. The second one was the statistical decision based on the bivariate multinomial model result with a value of *p* < 0.20 [16]. Thus, the multivariate multinomial model was composed of the predictors that met the assumptions and were adjusted together, without considering hierarchical order or determination level.

The backward stepwise procedure was used for the selection in the final model. The Likelihood Ratio Test (LRT) was used to test hypotheses about the significance of the predictor variables, that is, to evaluate the effect at all levels of the outcome simultaneously, which affects the number of parameters tested and the degrees of freedom associated with the test [46]. Thus, the comparison of the observed and the expected values using the likelihood function was based on the expression:

$$D=\;-2lnL_{reduced}-\left(-2lnL_{full)}\right)\sim X^{2}$$

The full model corresponds to the complete multivariate model and the reduced model to the model without a corresponding predictor, following a chi-square distribution with degrees of freedom equal to the number of set parameters (defined as zero under the null hypothesis—H0). Statistically significant results (*p* < 0.05) reject H0 and indicate that the predictor is significant for the model, being maintained in the final model.

## 3. Results

The socioeconomic and demographic characteristics, health status, HFSS, and aspects related to food handlers’ work in CR are in Table 1. The sample was composed mainly of women (62.2%), aged ≤40 years old (67.7%), with a partner (52.0%), and with up to eight years of education (54.1%). In most households, there were up to two people employed (78.8%), less than six rooms (64.4%), per capita household income below half a minimum wage (40%), and 19.3% of the workers in government programs. Among the health risk behaviors or conditions, overweight and alcohol use was more prevalent (60.8% and 46%, respectively) when compared to the smoking habit (17.2%) and the presence of NCDs (19.1%). More than half of these professionals were working in the CR for ≥12 months (57.3%) (Table 1). For most of the factors in Table 1, the median value was used to split the sample and perform the analysis.

Predictors of socioeconomic status (education, per capita household income, and participation in governmental programs) and geographic location of the CR were associated with the HFSS categories by the chi-square test (*p* < 0.05) (Table 1).

There was no significant association between mild FI and the demographic variables in the bivariate multinomial analysis. In contrast, the predictors of socioeconomic conditions were associated with both the mild and the moderate/severe FI category. Workers with less education were twice as likely to belong to the category with the highest severity of food insecurity (OR: 2.17; 95% CI: 1.25–3.77). Having lower per capita household income increased the chances of belonging to the category of mild insecurity by 86% (OR: 1.86; 95% CI: 1.20–2.88) and more than doubled the chance to be in the category of moderate/severe Insecurity (OR: 3.8; 95% CI: 2.18–6.60) in addition to participating in governmental programs (OR: 3.17; 95% CI: 1.75–5.74). Living in households with fewer rooms was also associated with the most severe food insecurity category (OR: 1.87; 95% CI: 1.05–3.34) (Table 2).

Predictors of health status, lifestyle, and work were not associated with any of the multinomial outcomes’ categories at a 5% significance level. However, working in the South, Southeast, or Midwest regions of Brazilian decreased the chances of belonging to one of the Food Insecurity categories between 21% to 42%, with significance only for the mild category (OR: 0.58; 95% CI: 0.38–0.88) (Table 2).

The bivariate multinomial regression models confirmed the associations of education, per capita income, participation in a governmental program, and the Brazilian region with the categories of HFSS, with an increase in the number of rooms among the predictors. Besides, variables that showed association with any of the categories of HFSS (*p*-value < 0.20) were also included in the multivariate multinomial model (gender, number of people working at home, and the presence of NCDs) (Table 2).

Table 3 shows the results of the multivariate model containing eight predictors selected in the previous steps. They were associated with at least one of the categories of HFSS with a value of *p* < 0.20. In the complete crude model, per capita income (Mild Insecurity—OR: 1.64; 95% CI: 1.01–2.66/Moderate/Severe Insecurity—OR: 2.7; 95% CI: 1.48–4.94); participation in a governmental program (Moderate/Severe Insecurity—OR: 2.02; 95% CI: 1.04–3.92) and CR region (Mild insecurity—OR: 0.5; 95% CI: 0.31–0.80) maintained an association with HFSS, increasing the chances of a CR worker belonging to one of the risk categories for food insecurity when in unfavorable socioeconomic situations, and compared to those with better socioeconomic conditions.

A stepwise backward method was applied to the multinomial model to adjust the final HFSS model. Calculating the odds ratios and standard errors and the model’s goodness adjustment indexes guided the model’s choice that best-suited data. Reduced models with smaller Akaike information criterion (AIC) than the previous model indicated the removal of the tested variable, as well as the *p*-value (>0.05) of the LRT (Table 4). The variables per capita household income, number of rooms, and number of people working (at the same home), in addition to the CR Brazilian region, remained in the model (Table 5).

Despite being maintained in the final model, considering the LRT results, the number of rooms and the number of people working did not maintain the association with food insecurity in the adjusted model. When tested, they were not confirmed (range of estimates < 10%). The multivariate model results confirmed the statistical significance of per capita household income as a predictor of HFSS. Food handlers with per capita household income equal to or less than half the minimum wage were 1.77 times more likely to be in a situation of mild food insecurity (OR: 1.77; CI95: 1.12–2.80) and around three times more likely to be in a situation of moderate/severe food insecurity (OR: 3.52; CI95: 2.00–6.19) when compared to individuals belonging to the food security category. In the adjusted model, the association with the CR Brazilian region was maintained in mild food insecurity. Therefore, the food handlers working in CR located in the Midwest/Southeast/South regions were 47% less likely to experience mild food insecurity (OR: 0.53; CI95: 0.34–0.83) compared to individuals in food security (Table 5).

## 4. Discussion

Food insecurity is a public health problem, affecting individuals in all parts of the world. The latest survey conducted in 2018 by the Food and Agriculture Organization of the United Nations (FAO) showed that about two billion people suffered from moderate or severe food insecurity, representing 26.4% of the world population [1]. In Latin America, including Brazil, 188 million people had the perception of moderate to severe food insecurity, representing 30.9% of the population [1]. In Brazil, the last populational study that assessed food insecurity in households was the National Household Sample Survey (PNAD/NHSS), carried out in 2013 and showed a prevalence of 22.6% of food insecurity in the population [36].

In Brazil, the foodservice market employs about 250 thousand food handlers [47], who work in several foodservice segments, such as this study’s locus (the CR). The findings can be extended to others in the foodservice segment, such as the food handlers working in hospitals, industrial or commercial food services. The activities of workers in the Brazilian foodservice sector revolve around a common goal, the production of meals for groups, whether healthy or sick [47], and, in the case of CR handlers, they provide meals for groups in social vulnerability [13]. The results of this study can be extended to the population of food handlers, given the peculiarities of this sector that include: (a) the composition of teams with professionals from different levels of educational background and with different and complementary activities [31]; (b) by workers from the different foodservice segments with a high prevalence of excess weight [30,32,48,49,50]; (c) by workers without professional training to carry out the activities, the in-service learning process takes place, which also leads to high turnover in the sector [31]; (d) inadequate work conditions, characterized by the requirement of long hours, rhythm and intense efforts with work overload, tasks such as repetitive movements, leading to absenteeism and high turnover of workers and the prevalence of work-related diseases [31]. This scenario reinforces the importance of the study and its scope.

Our results showed a total prevalence of 44.4% of food insecurity, much higher than the general population [51]. Moderate/severe food insecurity was almost twice the prevalence of the Brazilian population. In this study, socio-demographic characteristics showed that most food handlers were females in the age group ≤40 years old, similar to previous studies carried out with food handlers [15,16,18,22].

Two studies on food handlers from CR, the first in the city of Rio de Janeiro (*n* = 273 individuals from 7 CR) [21] and the second in the city of Belo Horizonte (*n* = 180 individuals from 4 CR) [22] showed a prevalence of total food insecurity of 53.7% and 24%, respectively. This prevalence, as in our study, is higher than food insecurity for the Brazilian population. It is worth mentioning that in these studies [21,22], most food handlers perceived themselves in mild food insecurity, according to the EBIA/BFIS classification [36]. There is a concern with access to food in the future, and it may impact the quality of the diet, choosing to buy cheaper foods [52].

In our study, an association was found between income and food insecurity, maintained in the regression model. Of the study participants, 39.9% had a per capita income at home equal to or less than half the minimum wage, classified as low-income families, according to the Brazilian Government [53]. Moreover, income showed a more significant impact on households with moderate to severe insecurity. Low-income families were about three times more likely to have food insecurity than workers with income above half the minimum wage. Other variables related to household income were associated with food insecurity as the number of rooms in the house and the number of individuals working, also maintained in the final regression model. Another variable related to household income that showed an association with food insecurity was the participation in a governmental program. Almost 40% of participants presented the requirements to receive governmental benefits [53], but only 19.3% of the participants received any benefit. Godoy et al. [20] also showed differences in food insecurity prevalence and income and education for CR customers. Among males, food insecurity prevalence was 64.2% when per capita income was below ¼ minimum wage and 54.8% from ¼ to half of the minimum wage. Females showed similar results, with 61.5% in food insecurity when per capita income was lower than ¼ minimum wage, and 54.8% when income was between ¼ to half of the minimum wage. CR customers with less than eight years of education also presented higher food insecurity for males and females (51% and 56.3%, respectively) [20].

In a previous study with the Brazilian population, 54.8% of households with moderate to severe food insecurity received per capita income up to half a minimum wage [51]. In the study carried out with food handlers in the CR in Rio de Janeiro, 59.4% of workers reported having another job to increase their income [21]. Per capita household income showed an inverse association with food insecurity in a study developed with food handlers in CR in Belo Horizonte/Brazil [22].

In a study that assessed food insecurity among workers in Canada, multivariate regression revealed that increased income independently decreases the chances of food insecurity in Canadian families, as well as having more workers in the family or at home. Income was considered a significant factor for the chances of food insecurity at home [24]. Some studies show an association between FI lower per capita income at home [53,54,55].

Schooling was associated with food insecurity at home in the bivariate analysis (*p* = 0.02), not remaining in the final regression model. In our study, 54.1% of the participants had an education level equal to or less than eight years, in agreement with other studies conducted with this professional category, identifying the highest percentage of individuals with educational level up to eight years of formal study (equivalent to elementary school) [19,21,34]. In general, education is associated with food insecurity among the general Brazilian population [51], in which a higher level of education relates to the lower prevalence of moderate or severe insecurity. Since food handlers in Brazil receive low salaries and present fewer years of formal education, they are more related to food insecurity even though working with food production.

In the study by Falcão et al. [21], an association was found between education and food insecurity in food handlers’ homes. In the studied sample, the risk of those who had up to nine years of formal study was almost three times more likely to find themselves in a food insecurity situation than those with higher education.

Another variable that showed association with food insecurity was the region of the CR. Food handlers from the Midwest-South regions perceived themselves in household food security. The National Household Sample Survey (PNAD/NHSS) [47] identified a higher prevalence of food insecurity in the North and Northeast regions (30%) than the Midwest/Southeast/South regions (less than 20%). Bezerra et al. [54] also demonstrated higher FI in the North (40.3%) and the Northeast (46.1%) with positive and moderate correlation between FI and the percentage of the extremely poor population. Gubert et al. [55] demonstrated a reduction in FI prevalence in all Brazilian states from 2004 and 2013, higher in the Northeast region. Santos et al. [56] evaluated the tendency and associated factors of food insecurity in Brazil, analyzing the NHSS from 2004, 2009, and 2013. These researchers verified that even though there was a reduction in food insecurity levels with time, there was an increase in the association force between food insecurity and regions North and Northeast of Brazil. Residents from these regions had three to four more chances of having moderate to severe food insecurity. With CR customers, only for females, there was an association between region and food insecurity, with higher prevalence in the north and northeast regions [20].

This study did not show an association between food security and excess weight, contrary to other studies [57,58,59,60] that showed an association between food insecurity and excess weight. Shamah-Levy et al. [60] associated food insecurity with undernutrition and obesity, discussing that both situations come from poor eating habits. In children, Kac et al. [58] discussed a less consistent association with overweight, but Franklin et al. [57] studying women presented a higher prevalence of excess weight with food insecurity. Godoy et al. [61] did not find a significant association between food insecurity and BMI or body fat percentage for CR’s male or female customers. Even though obesity is included in the NCDs, excess weight was not included as NCD for this study’s associations. There was an association between food insecurity and NCDs; however, it was not maintained in the model.

One of this study’s contributions is the confirmation of the dichotomy between FI and the performance in the food field. In the case of this study, places designed to promote access to food (CR) to prevent the occurrence of food insecurity also constitute a workspace for people in FI. Besides, the geographic location of the CRs act as protectors, confirming that regional inequalities in Brazil affect the same population group differently and confirming their claim that income is associated with FI. It is important to emphasize that the mean income from the North and Northeast regions is almost half of the income from workers from the South and Southeast regions in Brazil. This inequality is also observed in this food handler group and needs to be discussed inside a governmental program.

## 5. Conclusions

In Brazil, few studies assess workers’ food insecurity. Food handlers play an essential role in promoting food security as responsible for producing and supplying meals in hygienic and nutritional conditions suitable for the population using this state equipment. These workers have characteristics, confirmed in the study, as low education and low income, which places them both as actors and target population of public policies aimed at food insecurity. This study is the first to assess food handlers nationwide, and a high prevalence of food insecurity was observed, higher than that of the general population. For moderate/severe food insecurity, it was almost twice the Brazilian population. Based on the study design, it is not possible to establish a causal link between the variables, but the study shows paths for further studies. The variables that showed an association for this population were the household income per capita for the different levels of food insecurity and the CR region for mild food insecurity. The high prevalence of food insecurity in this population and the return of the increase in the prevalence of food insecurity in the Brazilian and worldwide population point to the need for further studies on the theme with categories of workers, mainly low-income, which allow identifying factors related to the work environment and developing policies and interventions aimed at preventing food insecurity and its consequences for health.

## Figures and Tables

**Table 1:** Descriptive analysis of the study population, considering the distribution in Household Food Security Situation of demographic, socioeconomic characteristics, health status and lifestyle, and work-related aspects.

Variables	% (*n*)	Food Security % (*n*)	Food Insecurity		*p*-Value *
			Mild % (*n*)	Moderate/Severe % (*n*)	
Gender (*n* = 471)					0.44
Female	62.2 (293)	53.9 (158)	29.01 (85)	17.06 (50)	
Male	37.8 (178)	58.4 (104)	28.65 (51)	12.92 (23)	
Age group (*n* = 471)					0.56
≤40 years old	67.7 (319)	54.23 (173)	30.41 (97)	15.36 (49)	
>40 years old	32.3 (152)	58.55 (89)	25.66 (39)	15.79 (24)	
Education level (*n* = 471)					0.02
≤08 years of study	54.1 (255)	51.37 (131)	29.02 (74)	19.61 (50)	
>08 years of study	45.9 (216)	60.65 (131)	28.70 (62)	10.65 (23)	
Marital status (*n* = 471)					0.43
With partner	52.0 (245)	55.1 (135)	31.02 (76)	13.88 (34)	
Without a partner	48.0 (226)	56.2 (127)	26.55 (60)	17.26 (39)	
Number of workers in the family (*n* = 471)					0.15
1 or 2 work	78.8 (364)	53.8 (196)	28.85 (105)	17.31 (63)	
Above 2 workers	21.2 (98)	63.3(62)	26.53 (26)	10.2 (10)	
Income (per capita minimum wage) ^1^ (*n* = 451)					0.00
>1/2 MW	60.1 (271)	64.6 (175)	25.8 (70)	9.6 (26)	
≤1/2 MW	39.9 (180)	43.3 (78)	32.22 (58)	24.44 (44)	
Governmental program participation (*n* = 471)					0.00
No	80.7 (380)	59.2 (225)	28.16 (107)	12.63 (48)	
Yes	19.3 (91)	40.7 (37)	31.87 (29)	27.47 (25)	
Number of rooms at home (*n* = 463)					0.07
<6 rooms	35.6 (165)	61.8 (102)	26.67 (44)	11.52 (19)	
≥6 rooms	64.4 (298)	52.0 (155)	29.87 (89)	18.12 (54)	
Health status and lifestyle					
NCD (n = 471)					0.19
Yes	19.1 (90)	62.2 (56)	20.0 (18)	17.78 (16)	
No	80.9 (381)	54.1 (206)	30.97 (118)	14.96 (57)	
Weight excess (*n* = 469)					0.52
Yes	60.8 (285)	53.7 (153)	29.82 (85)	16.49 (47)	
No	39.2 (184)	58.7 (108)	27.72 (51)	13.59 (25)	
The habit of drinking alcohol (*n* = 470)					0.99
No	54.0 (254)	55.5 (141)	29.13 (74)	15.35 (39)	
Yes	46.0 (216)	55.6 (120)	28.70 (62)	15.74 (34)	
Smoking habit (*n* = 470)					0.25
No	82.8 (389)	55.0 (214)	30.33 (118)	14.65 (57)	
Yes	17.2 (81)	58.0 (47)	22.22 (18)	19.75 (16)	
Work-related aspects					
Time working at the CR (*n* = 471)					0.58
<12 months	42.7 (201)	58.2 (117)	27.86 (56)	13.93 (28)	
≥12 months	57.3 (270)	53.7 (145)	29.63 (80)	16.67 (45)	
Management model (*n* = 471)					0.79
Direct management	38.0 (179)	53.6 (96)	30.17 (54)	16.2 (29)	
Outsourced	62.0 (292)	56.8 (166)	28.1 (82)	15.07 (44)	
Brazilian region of CR (*n* = 471)					0.03
North/Northeast	45.7 (215)	49.8 (107)	34.42 (74)	15.81 (34)	
Midwest/South/Southeast	54.4 (256)	60.6 (155)	24.22 (62)	15.23 (39)	

* Pearson’s chi-square *p*-value. ^1^ Minimum wage (MW): 175.90 USD per month.

**Table 2:** Bivariate multinomial logit model with odds ratio (OR) estimation between household food security situation (reference group: food security) and demographic, socioeconomic, health, lifestyle predictors, and those related to work in a Community Restaurant in all the Brazilian regions.

Predictor Variables	Mild Insecurity				Moderate/Severe Insecurity			
	OR	SE	*p*-Value	IC95%	OR	SE	*p*-Value	IC95%
**Gender (*n* = 471)**								
Female	1.1	0.24	0.67	0.72–1.68	1.43	0.4	0.20	0.82–2.49
**Marital status (*n* = 471)**								
Without partnet	0.84	0.18	0.41	0.55–1.27	1.22	0.32	0.45	0.73–2.05
**Age group (*n* = 471)**								
>40 years old	0.78	0.18	0.28	0.50–1.23	0.95	0.27	0.86	0.55–1.65
**Education level (*n* = 471)**								
Elementary Education (complete/incomplete)	1.19	0.25	0.40	0.79–1.81	2.17	0.61	0.00	1.25–3.77
**Income (per capita minimum wage) (*n* = 451)**								
≤1/2 MW	1.86	0.42	0.00	1.20–2.88	3.8	1.07	0.00	2.18–6.60
**Number of rooms**								
<6 rooms	1.33	0.30	0.20	0.86–2.07	1.87	0.55	0.03	1.05–3.34
**Number of workers in the family**								
1 or 2 work	1.28	0.34	0.35	0.76–2.14	1.99	0.74	0.06	0.96–4.12
**Governmental program participation (*n* = 471)**								
Yes	1.65	0.45	0.07	0.96–2.82	3.17	0.96	0.00	1.75–5.74
Health status and lifestyle								
**Weight excess (*n* = 469)**								
Yes	1.18	0.26	0.45	0.77–1.80	1.33	0.37	0.31	0.77–2.29
**NCD (*n* = 471)**								
No	1.78	0.52	0.05	1.00–3.17	0.97	0.31	0.92	0.52–1.82
**Smoking habit (*n* = 470)**								
Yes	0.69	0.21	0.22	0.39–1.25	1.28	0.42	0.45	0.68–2.42
**The habit of drinking alcohol(*n* = 470)**								
Yes	0.98	0.21	0.94	0.65–1.49	1.02	0.27	0.93	0.61–1.72
Work aspects								
**Time working at CR (*n* = 471)**								
≥12 months	1.15	0.25	0.51	0.76–1.75	1.3	0.35	0.34	0.76–2.21
**Management model (*n* = 471)**								
Outsourced	0.88	0.19	0.55	0.57–1.34	0.88	0.24	0.63	0.52–1.49
**Brazilian region (*n* = 471)**								
Midwest/South/Southeast	0.58	0.12	0.01	0.38–0.88	0.79	0.21	0.381	0.47–1.33

OR: Odds ratio; SE: standard error. * *Chi*-Square test statistic *p*-value. ^1^ Minimum wage (MW): 175.90 USD per month.

**Table 3:** Multivariate multinomial logit model for predictors of Household Food Security Situation (reference group: food security) in food handlers of community restaurants.

Variables	Mild Insecurity				Moderate/Severe Insecurity			
	OR	SE	*p*-Value	IC95%	OR	SE	*p*-Value	IC95%
Incomplete/complete elementary education	1.27	0.3	0.32	0.79–2.02	1.76	0.54	0.07	0.96–3.22
Gender: Female	1.27	0.31	0.32	0.79–2.05	1.23	0.38	0.50	0.67–2.26
Income: ≤1/2 MW	1.64	0.4	0.04	1.01–2.66	2.7	0.83	0.001	1.48–4.94
Number of rooms ≥ 6	0.83	0.20	0,45	0.52–1.34	0.65	0.21	0,18	0.35–1.22
1 or 2 work in the family	1.02	0.29	0.93	0.59–1.79	1.22	0.48	0.61	0.56–2.64
Government program participation: Yes	1.29	0.39	0.41	0.71–2.34	2.02	0.68	0.04	1.04–3.92
NCD: No	1.62	0.51	0.12	0.88–2.98	1.07	0.38	0.85	0.53–2.15
Brazilian region: Midwest/South/Southeast	0.5	0.12	0.004	0.31–0.80	0.68	0.2	0.20	0.38–1.22

OR: Odds ratio; SE: standard error.

**Table 4:** Comparison of the complete and incomplete model performed using the Likelihood Ratio Test (LRT)—stepwise backward method.

LRT (Complete and Incomplete Model)	Obs.	ll(Nulo)	ll (Model)	gl	AIC	BIC	X^2^ (LR X^2^ (2))	p > X^2^
Complete Model backward	439	−427.5	−402.8	18	841.70	915.20	49.31	0.00
Model without education	439	−427.49	−404.65	16	841.296	906.649	3.63	0.16
Model without education and gender	439	−427.49	−405.11	14	838.211	895.394	4.54	0.34
Model without education, gender, and income	458	−447.10	−431.27	12	886.54	936.066	56.88	0,00
Model without education, gender, and number of workers	443	−431.19	−409.68	12	843.359	892.48	13.69	0.03
Model without education, gender, and government program	439	−427.49	−407.51	12	839.028	888.042	9.36	0.15
Model without education, Gender, government program, and number of rooms	443	−430.49	−412.33	10	844.652	885.588	18.98	0.01
Model without education, Gender, government program, and Region	439	−427.49	−411.20	10	842.407	883.252	16.74	0.03
Model without education, Gender, government program, and NCD	439	−427.49	−408.64	10	837.273	878.118	11.61	0.17

LRT: Likelihood Ratio Test; ll = Log likelihood; gl = degrees of freedom; AIC = Akaike information criterion; BIC = Bayesian information criterion.

**Table 5:** An adjusted multivariate model of the Household Food Security Situation (HFSS) (reference group: food security) determinants among food handlers in Brazilian community restaurants.

HFSS Model Adjusted	Mild Insecurity				Moderate/Severe Insecurity			
	OR	SE	*p*-Value	IC95%	OR	SE	*p*-Value	IC95%
Income ≤ 1/2 MW	1.77	0.41	0.02 *	1.12–2.80	3.52	1.02	0.00 *	2.00–6.19
Number of rooms < 6	0.77	0.18	0.27	0.48–1.23	0.58	0.18	0.08	0.31–1.07
Number of workers: 1 or 2	1.08	0.30	0.79	0.62–1.87	1.37	0.53	0.42	0.64–2.92
Brazilian region: Midwest/South/Southeast	0.53	0.12	0.01 *	0.34–0.83	0.76	0.21	0.32	0.43–1.32

Model adjusted for income, number of rooms, and workers in the household and region of the CR. 95% CI: 95% confidence interval; OR: Odds Ratio; SE: standard error. * significance level *p* < 0.05.

## Data Availability

Data sharing is not applicable to this article.
